# Chronic Artificial Blue-Enriched White Light Is an Effective Countermeasure to Delayed Circadian Phase and Neurobehavioral Decrements

**DOI:** 10.1371/journal.pone.0102827

**Published:** 2014-07-29

**Authors:** Raymond P. Najjar, Luzian Wolf, Jacques Taillard, Luc J. M. Schlangen, Alex Salam, Christian Cajochen, Claude Gronfier

**Affiliations:** 1 Inserm U846, Stem Cell and Brain Research Institute, Bron, France; 2 University of Lyon, Claude Bernard Lyon 1, Villeurbanne, France; 3 Wolf Technologieberatung - Object-Tracker, Perchtoldsdorf, Austria; 4 University of Bordeaux, Sommeil, Attention et Neuropsychiatrie, USR 3413, Bordeaux, France; 5 CNRS, Sommeil, Attention et Neuropsychiatrie, USR 3413, Bordeaux, France; 6 Philips Research, High Tech Campus 34 5656AE, Eindhoven, The Netherlands; 7 Institut Polaire Français Paul-Emile Victor (IPEV), Technopôle Brest-Iroise - BP 75, Plouzané, France; 8 Centre for Chronobiology Psychiatric University Clinic, University of Basel, Basel, Switzerland; University of Texas Southwestern Medical Center, United States of America

## Abstract

Studies in Polar Base stations, where personnel have no access to sunlight during winter, have reported circadian misalignment, free-running of the sleep-wake rhythm, and sleep problems. Here we tested light as a countermeasure to circadian misalignment in personnel of the Concordia Polar Base station during the polar winter. We hypothesized that entrainment of the circadian pacemaker to a 24-h light-dark schedule would not occur in all crew members (n = 10) exposed to 100–300 lux of standard fluorescent white (SW) light during the daytime, and that chronic non-time restricted daytime exposure to melanopsin-optimized blue-enriched white (BE) light would establish an a stable circadian phase, in participants, together with increased cognitive performance and mood levels. The lighting schedule consisted of an alternation between SW lighting (2 weeks), followed by a BE lighting (2 weeks) for a total of 9 weeks. Rest-activity cycles assessed by actigraphy showed a stable rest-activity pattern under both SW and BE light. No difference was found between light conditions on the intra-daily stability, variability and amplitude of activity, as assessed by non-parametric circadian analysis. As hypothesized, a significant delay of about 30 minutes in the onset of melatonin secretion occurred with SW, but not with BE light. BE light significantly enhanced well being and alertness compared to SW light. We propose that the superior efficacy of blue-enriched white light versus standard white light involves melanopsin-based mechanisms in the activation of the non-visual functions studied, and that their responses do not dampen with time (over 9-weeks). This work could lead to practical applications of light exposure in working environment where background light intensity is chronically low to moderate (polar base stations, power plants, space missions, etc.), and may help design lighting strategies to maintain health, productivity, and personnel safety.

## Introduction

Amongst other possible *zeitgebers* such as food, exercise and temperature, light is the strongest synchronizer of the circadian timing system in humans [1]–[3]. An inappropriate light signal leads to circadian misalignment, and neurocognitive, temperature, cardiovascular, immunological, sleep, vigilance and memory alterations [4]–[10]. The entraining effect of light is dependent upon timing [11], intensity [12], [13], duration [3], [14], wavelength [15]–[17] and pattern [10], [18]–[20] of the exposure. Therefore, optimization of one or many of these parameters could favor entrainment of the circadian system.

Peak circadian sensitivity to light is shifted towards the blue region of the spectrum (460 to 480 nm) [15]–[17], [21], and corresponds to the recently discovered non-visual melanopsin-based photoreceptive system [22]–[24]. Together with the conventional visual photoreceptors (rods, cones), the melanopsin system conveys photic information to non-visual brain structures such as the biological clock in the suprachiasmatic nuclei (SCN) to allow circadian photo-entrainment [25]–[28].

Success of long duration space and polar missions depend on a finely entrained circadian system, which can only be achieved with optimal background illumination. Optimal lighting conditions, however, are often lacking in extreme environments. During winter, crew members of polar base stations are utterly deprived of natural sunlight and rely solely on artificial illumination. Physiological disorders have been reported in some individuals during the polar winter. Alterations include circadian misalignment, subjective and sleep structure alterations and even free-running [2], [29]–[35]. In 2008 Francis and colleagues found that increasing environmental light intensity significantly enhanced personnel light exposure, however, it did not correct for (or avoid) the polar winter delays in sleep [36].

High color temperature lamps contain more energy in the blue range of the light spectrum than conventional lamps, and are expected to optimize stimulation of the human circadian system. Compared to lower color temperatures (3000 K), higher color temperature lighting has indeed been shown to increase mental activity at 1000 lux [37], enhance the central nervous system activity [38] and decrease drowsiness at intensities as low as 30 lux [39]. Moreover, blue-enriched light in the workplace has been found to enhance subjective sleep quality, subjective alertness and subjective performance compared to regular white light [40]. Whether light strategies can have long term and sustained effects on the circadian system, mood and cognitive performance is, however, unknown. Thus, in the present study, we investigate the chronic effect of blue-enriched white (BE) light (17000K) *versus* standard white (SW) light (4100 K) on circadian entrainment, rest activity cycle, mood and neurobehavioral performances in the crew members of the scientific Concordia polar base station (South Pole) during winter.

## Materials and Methods

### Participants

The study took place during winter time, between February and November 2009, at the Concordia base station (DOME C, 75° S 123° E, South Pole). Ten crew members (8 males, 2 females, 30±2.1 years) participated in this 9 week study (May - July 2009). The protocol, questionnaires, and consent form were approved by the Institutional Review Board of the University of Basel (EKBB/Ethikkommission beider Basel, Switzerland) and were in agreement with the Declaration of Helsinki. All participants gave written informed consent, and the Base doctor attested to their fitness to take part in the study. Participants filled the Morningness-Eveningness Questionnaire (MEQ) [41], a health questionnaire, the Pittsburgh Sleep Quality Inventory (PSQI) [42], and the Beck Depression Inventory (BDI) [43] prior to study start.

### The Antarctica station

The Concordia Research Station is a research facility located on the continental Antarctic plateau, at an altitude of 3,233 m above sea level, 75° 06 'S and 123° 21 'E, at a location called Dome C. Above the Polar Circle latitudes of 66.33′, north or south, the sun is above the horizon for 24 hours for at least 1 day per year (mid-summer day, midnight sun) and concomitantly below the horizon for 24 hours for at least 1 day per year (midwinter day). At Concordia (75°S), the sun does not rise for ∼100 days in the winter (May 4 - August 10, 2009) and does not set for ∼105 days in summer (before February 2, and after October 30, 2009). During summer in Polar Regions, the outdoor light intensity can exceed 40 000 lux, however, during the winter with only artificial light, and an orange glow on the horizon at noon, the maximum light exposure possible is around 500–700 lux [2], [36]. During summer, blinds of the station could be closed or glasses could be worn to mimic a light-dark cycle. On the other hand, during the winter period, when the sun is below the horizon, the strength of the light-dark cycle as a circadian synchronizer is very low and possibly insufficient (For review, see [44]).

### Daily work routines of the participants

This study was conducted in a polar station where our study participants were workers on a mission in the Antarctic. Subjects we assigned to different tasks in the Concordia base station, according to their roles. Their work-schedule was based on a regular daytime work; 6 days a week, with no shiftwork, and no extended hours (8–10 h a day on average). They were encouraged to go to bed at their habitual bedtimes (habitual sleep times and habitual wake times), but were not constrained to retire at a fixed time. As in real life, most participants used an alarm clock in the morning to comply with their work schedule. Subject's behavior, sleep times, and light exposure were monitored as indicated below.

### Light intervention

**Figure 1:**
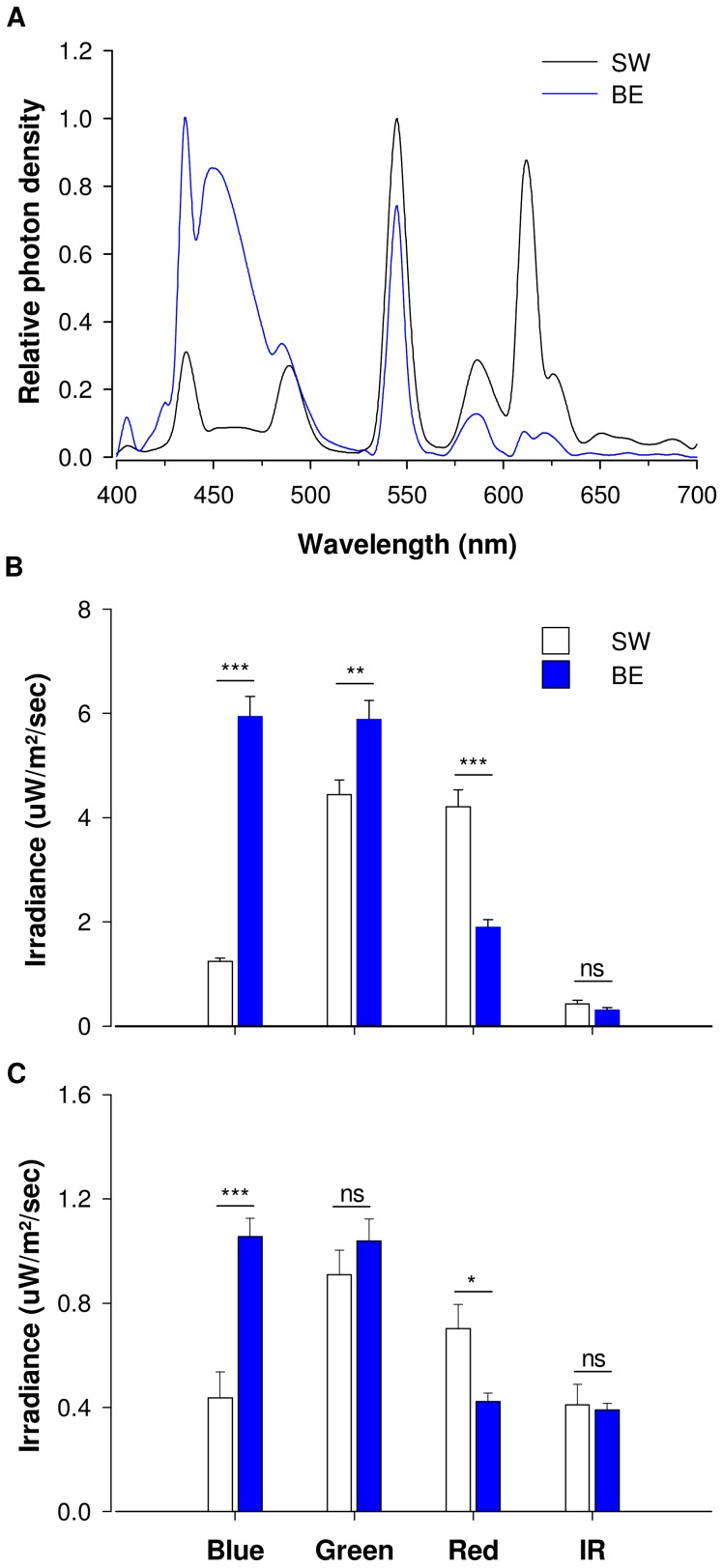
Characteristics of the lighting environments. **A**. Spectra of ambient lightings used in the study. In a black straight line the spectrum of the control SW light used in weeks 1, 4, 5, 8 and 9 of the study. In a blue straight line the spectrum of the BE light used in weeks 2, 3, 6, and 7 of the study. **B**. Intensity (irradiance) and spectral composition of the lighting environments as measured in different locations of the Polar Station. BE light contained significantly more energy in the blue (p<0.001) and green (p<0.01) range of the light spectrum, and less red (p<0.001) compared to SW light. **C**. Intensity (irradiance) and spectral composition of the lighting environments as measured by the Lightwatcher device worn by the participants. The light spectra measured by the polar station workers under BE light contained significantly more short wavelength blue light (p<0.001) and less long wavelength red light (p<0.05) compared to SW light. Intensity of middle wavelength green light and IR light detected by the participants were not significantly different. See Table S1 and Figures S2 and S3 for more details.

Indoor light was customized throughout the 9 weeks of the study. Participants were in SW light (correlated color temperature of 4,100 K) during the starting week 1 (SW1), which was considered as baseline. Lighting conditions after SW1 were alternated every 2 weeks. During weeks 2, 3, 6 and 7 subjects were exposed to BE light (Philips, ActiViva Active, TLD 36 W, correlated color temperature of 17,000K) during the daytime, whereas during weeks 4, 5, 8 and 9 they were exposed to SW light (Table 1). Light bulbs in strategically chosen areas of the habitat (e.g. in restaurant/galley, recreational quarters, living quarters, where subjects spent most of their time) were replaced every 2 weeks, alternating between SW fluorescent light bulbs and high color temperature/blue-enriched fluorescent lights bulbs (Figure 1. A).

**Table 1:** Protocol of the study.

Weeks	1	2	3	4	5	6	7	8	9
**Lighting condition**	SW	BE	BE	SW	SW	BE	BE	SW	SW
**Actigraphy**	+	+	+	+	+	+	+	+	+
**Light sensor**	+	+	+	+	+	+	+	+	+
**Salivary melatonin (samples)**	8	8	8	8	8	8	8	8	8
**Neurobehavioral test battery**	+	-	+	-	+	-	+	-	+

+: monitoring.

-: not monitoring.

After one baseline week (SW1), lighting conditions were modified every 2 weeks for 9 weeks. Participant's sleep-wake rhythm and personal light environment were continuously monitored throughout the study via actigraphy and light sensors, respectively. Eight saliva samples were collected on one day every week (6 samples before bedtime and 2 samples after wake time) and a neurobehavioral test battery was performed at the end of each lighting block.

### Monitoring of individual spectral light exposure

Spectral composition and intensity were measured in 97 locations of the station (labs, kitchen, exercise room, bedrooms …). Eight measures were made per location with a different angle of gaze (right, left, forward, backward) and position (sitting up, standing up, walking and walking up). Individual spectral light exposure of the participants was measured in 5 spectral bands: IR (860 nm), red (620 nm), green (540 nm), blue (460 nm) and UV light (350 nm) (Figure S1). Light irradiance measurements were implemented with a newly developed personal data logger (LightWatcher, Wolf Technologieberatung - Object-Tracker, Perchtoldsdorf, Austria) worn by subjects (Headset or glasses or by badge). The personal data logger is very compact with dimensions of 10 mm×20 mm×50 mm (USB-stick size), light-weight with 10 grams mass, battery operated, USB-2 interface, and measures irradiance at 5 wavelength bands, along with the ambient temperature, humidity, barometric pressure and acceleration in 3 directions.

### Monitoring of sleep duration and sleep quality

Sleep duration and sleep quality were estimated using actigraphy watches (Actitracs, IM Systems, USA, or Actiwatch L, Cambridge Neurotechnology) continuously worn by each subject around the wrist of the non-dominant arm. Subjects were also instructed to fill out a sleep diary from which lights off time, bed time and wake time data were collected.

### Monitoring of alertness and neurobehavioral performance

A neurobehavioral test battery was performed on the 5^th^ day (Friday) of the second week of a light exposure, in the evening (at 18:00h or 20:00h). Test battery included a computerized questionnaire on self-assessments of alertness, mood, sleepiness (Karolinska Sleepiness Scales (KSS)), motivation, well being measures and a reaction time task (Psychomotor vigilance performance (PVT)).

### Monitoring of circadian entrainment

Circadian phase was monitored via salivary melatonin secretion. Saliva was sampled on the 5^th^ day (Friday) of each week at hourly intervals during the 5 hours preceding bedtime (6 samples), and during the first hour after wake time (2 samples), all in a dimly lit environment (1–20 lux). Meals were scheduled after saliva collection or at least 30 minutes prior to a collection to avoid contamination of the samples. After meals, teeth were brushed with water only. Saliva (1 ml) was collected by placing an absorbing swab into the mouth and moving it around slowly to collect saliva during 5 minutes (during this time the psychometric tests were filled out). Next, the swab was deposed in a salivette tube. The samples were kept in the refrigerator at 4°C until the end of the day, and then frozen and stored at −20°C, until assayed. Salivary melatonin was assayed in our laboratory using the Bühlmann Direct Saliva Melatonin Radioimmunoassay (RIA) Kits (Bühlmann AG, RK-DSM2, Switzerland) based on the Kennaway G280 anti-melatonin antibody. Melatonin RIA had an analytical sensitivity of 0.2 pg/ml and a functional sensitivity of 0.9 pg/ml. Intra-assay coefficient of variation was 7.5% at 1.9 pg/ml and 4.5% at 24 pg/ml (n = 16). Inter-assay coefficient of variation was 10.1% at 1.9 pg/ml and 8.1% at 24 pg/ml (n = 16). Dim light melatonin onset (DLMO) was estimated for each subject at the end of each 2 week light segment.

### Data and statistical analyses

Statistical analyses were performed using Statistica (StatSoft, Inc., Tulsa, OK, USA). Light intensities and spectral composition percentages were compared between the two lighting conditions using a t-test for independent variables. Irradiance and spectral light composition across BE and SW weeks were compared using one-way ANOVA with time as a repeated factor. Actigraphy data were analyzed using the non-parametric circadian analysis procedure (NPCA) [45] on 7-days segments over all 9 weeks. In this analysis, intra-daily stability (IS) is an indicator of stability of the rest activity cycle; inter-daily variability (IV) is a marker of fragmentation of activity; and relative amplitude is a measure of amplitude of the rest-activity cycle. DLMOs were determined using an individual threshold defined as the mean plus two standard deviations of the three lowest consecutive daytime points (method derived from [46]). In order to correct for individual initial conditions at the beginning of the study, DLMOs, actigraphy, questionnaires and sleep timing data were expressed relative to week 1 (SW1) original values (Gronfier et al. 2007). DLMOs, actigraphy parameters, questionnaires data and sleep timing data of the second light exposure week were pooled based on lighting condition and compared using a Wilcoxon Matched Pairs Test. A binomial test was used to determine whether light condition had a random effect on the direction of phase shift (null hypothesis: 50% phase advances, 50% phase delays), Neurobehavioral data were compared using a general linear model GLM, with time and light condition as two repeated factors. Data are expressed as mean ± SE.

## Results

### Lighting characteristics

Illuminances measured at ∼1.80 m in the average horizontal angle of gaze were not significantly different between lighting conditions during daytime: 160 lux (6.5–470 lux) under SW light and 171 lux (28–369 lux) under BE light (see Table S1 for details). Alternation of the lighting environment between SW and BE was successful and closely monitored (Figure S2). SW light's spectral composition measured at different locations of the station was on average: 12% of blue light, 43% of green light, 41% of red light, and 4% of IR light (Figure S3). BE light's spectral composition was on average: 42% of blue light, 42% of green light, 14% of red light, and 2% of IR light (Figure S3). Based on environmental light measurements made in different locations of the polar station, BE light contained 376% and 33% more energy in the blue (p<0.001) and green (p<0.01) range of the light spectrum respectively, and 55% less energy in the red (p<0.001) range of light spectrum, compared to SW light (Figure 1. B). There was no significant difference in the IR content of both lighting environments. Based on individual, proxy estimates of corneal light intensity, participants received significantly 142% more blue light (p<0.001) and 40% less red light (p<0.05) during BE light condition compared to SW condition (Figure 1. C, Figure S2). There was no difference in the intensity of green or IR light received by the participants under SW and BE light conditions. There was no significant difference in irradiance and spectral light composition across BE weeks nor across SW weeks. UV radiation, usually from natural sunlight, was below the detection limit of the LightWatcher.

### Actigraphy results

**Figure 2:**
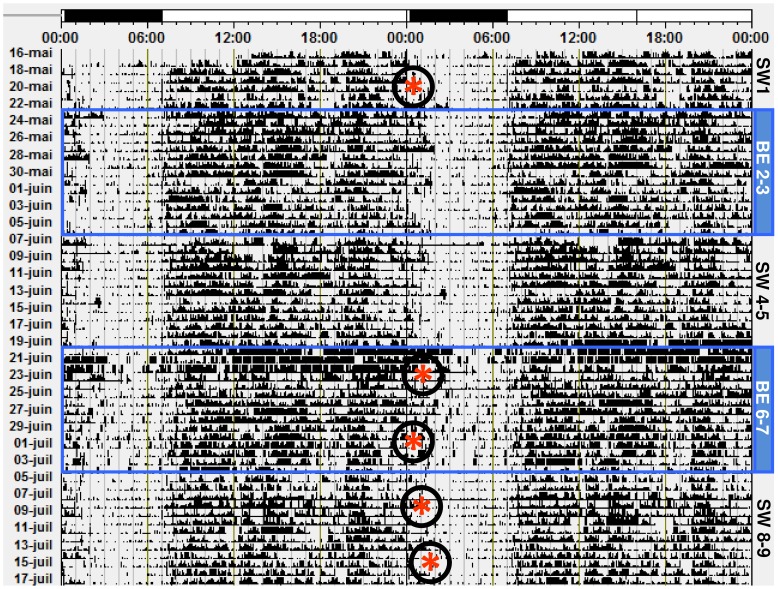
Representative raster plot of continuous actigraphy over 9 weeks, and circadian phase. Subject (S7) had a relatively regular sleep wake cycle across the studyCircadian phase, assessed by DLMO (*), shows a phase delay under SW light condition (SW 8–9), but not under BE light condition (BE 6–7), compared to W1.

Actigraphy data are illustrated in a double raster plot. All subjects maintained a regular sleep wake cycle and showed relative phase stability (Figure 2). Some subjects showed phase changes in activity, and this, independently of lighting conditions (Figure S9, Figure S10). NPCA measures, such as IS, IV, and amplitude were not significantly different between light conditions (p = ns). As illustrated in Figure S4, average activity and its temporal profile was not significantly different between the 2 light conditions (340±31 counts/24 h, 348±39 counts/24 h during SW and BE light weeks respectively, p = 0.4).

#### Sleep results

**Figure 3:**
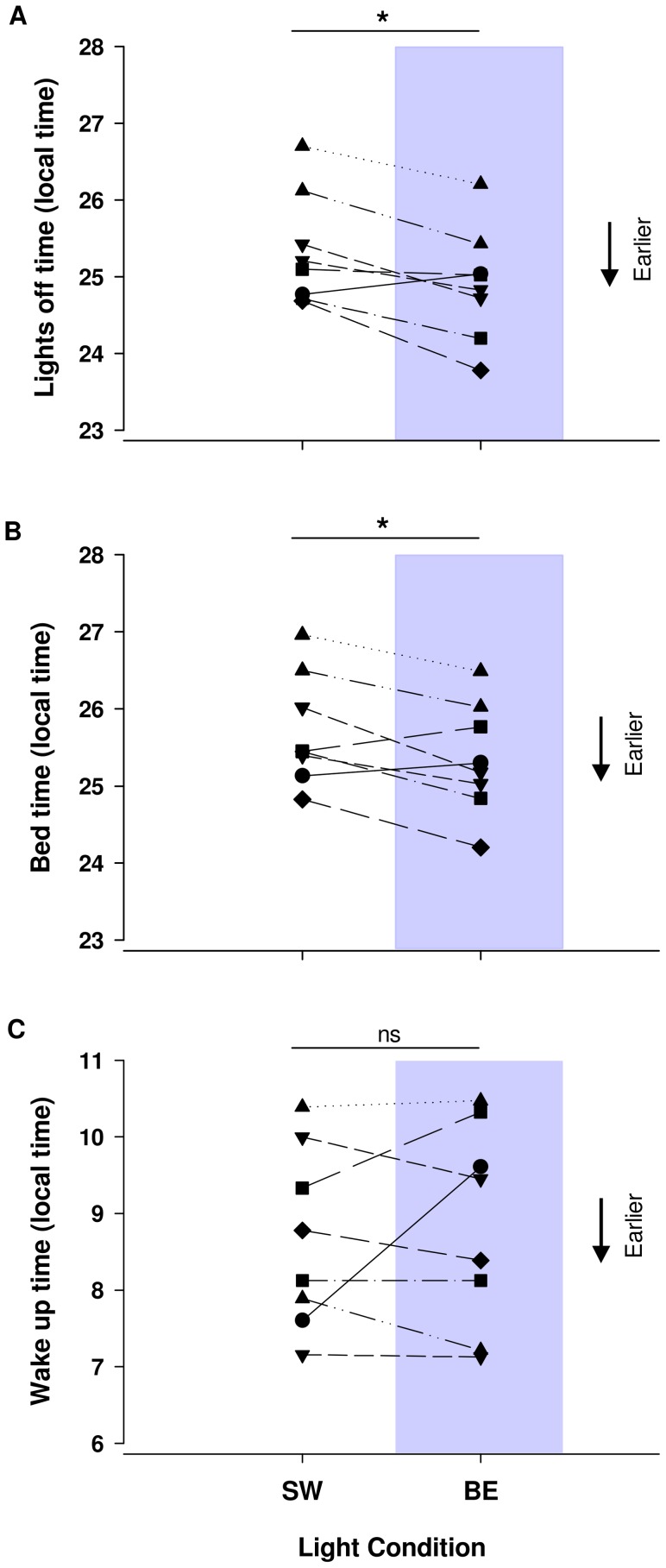
Impact of SW and BE light on the participants' lights off time and sleeping pattern. Average lights off time (**A**) and bed time (**B**) were significantly earlier under BE light weeks (24.9±0.3 h and 25.4±0.3 h, respectively) in comparison SW light weeks (25.3±0.3 h and 25.7±0.3 h, respectively) (p<0.05). Wake up time was not significantly different between lighting conditions, 9.0±0.6 h and 8.6±0.4 h under BE and SW light respectively (p = 0.4).

Lights off time, bed time and wake time data were successfully collected from eight participants' sleep diaries. There was a significant delay in bedtime (p<0.05) and time of lights off (p<0.05) under SW light weeks compared to BE light weeks (Figure 3. A, B, and Table 2). Wake up time on the other hand was not significantly different between these two lighting conditions (p = 0.6) (Figure 3. C). As a result, there was a significant increase in sleep duration during BE light weeks compared to SW light weeks (7∶27±0∶24, 6∶45±0∶30, p<0.05). Sleep efficiency derived from actigraphy was not different between light conditions (77.7±5.1%, 78.7±5.3% during SW and BE light weeks respectively) (Table 2).

**Table 2:** Summary of sleep and phase shift results.

	SW	BE	Statistics#
**Lights off time**	1∶20±0∶15	0∶54±0∶15	p<0.05
**Bed time**	1∶43±0∶15	1∶21±0∶15	p<0.05
**Wake up time**	8∶39±0∶24	8∶50±0∶27	ns
**Sleep duration**	6∶45±0∶30	7∶27±0.23	p<0.05
**Sleep efficiency (%)**	77.7±5.1	78.7±5.3	ns
**Phase delay (DLMO)**	0∶31±0∶12	0∶02±0∶12	p<0.05
**Phase angle** *	1∶51±0∶21	1∶40±0∶06	ns

Values are averages±SEM.

# Statistics computed using the Wilcoxon matched pairs test.

*Phase angle  =  Sleep onset time - DLMO time.

All times are given in hh:mm.

In coherence with a significantly later circadian phase, lights off time, bed time were significantly delayed under SW light weeks in comparison to BE light weeks. On the other hand wake up time was not significantly different between lighting conditions, this leading to a significant decrease in sleep duration under SW light weeks. Sleep efficiency was not different between lighting conditions. Phase angle between DLMO and lights off was not different between lighting conditions.

#### Circadian phase results

**Figure 4:**
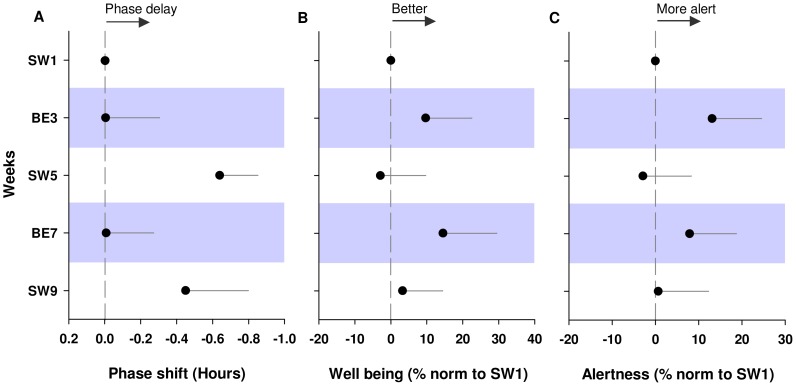
Circadian and neurobehavioral changes under SW and BE light condition over 9 weeks. Results were normalized to baseline SW1. **A**. Circadian phase was assessed using salivary DLMO. A delay in circadian phase of −0.64±0.21 h and −0.45±0.34 h was observed during weeks W5 and W9 respectively under SW light (p<0.05). The circadian phase delay observed on week W5 appears to be corrected during BE light on week BE7, and is observed again on week SW9 under SW light. **B**. Well being was increased by BE light (+9.7±12.8% and +14.5±14.9% on BE3 and BE7 respectively) compared to SW light (−2.9±12.8% and +3.2±11.2% during SW5 and SW9 respectively) (p<0.05). **C**. Subjective alertness was marginally increased on weeks BE3 and BE7 under BE light (p = 0.08) compared to SW light weeks SW5 and SW9.

**Figure 5:**
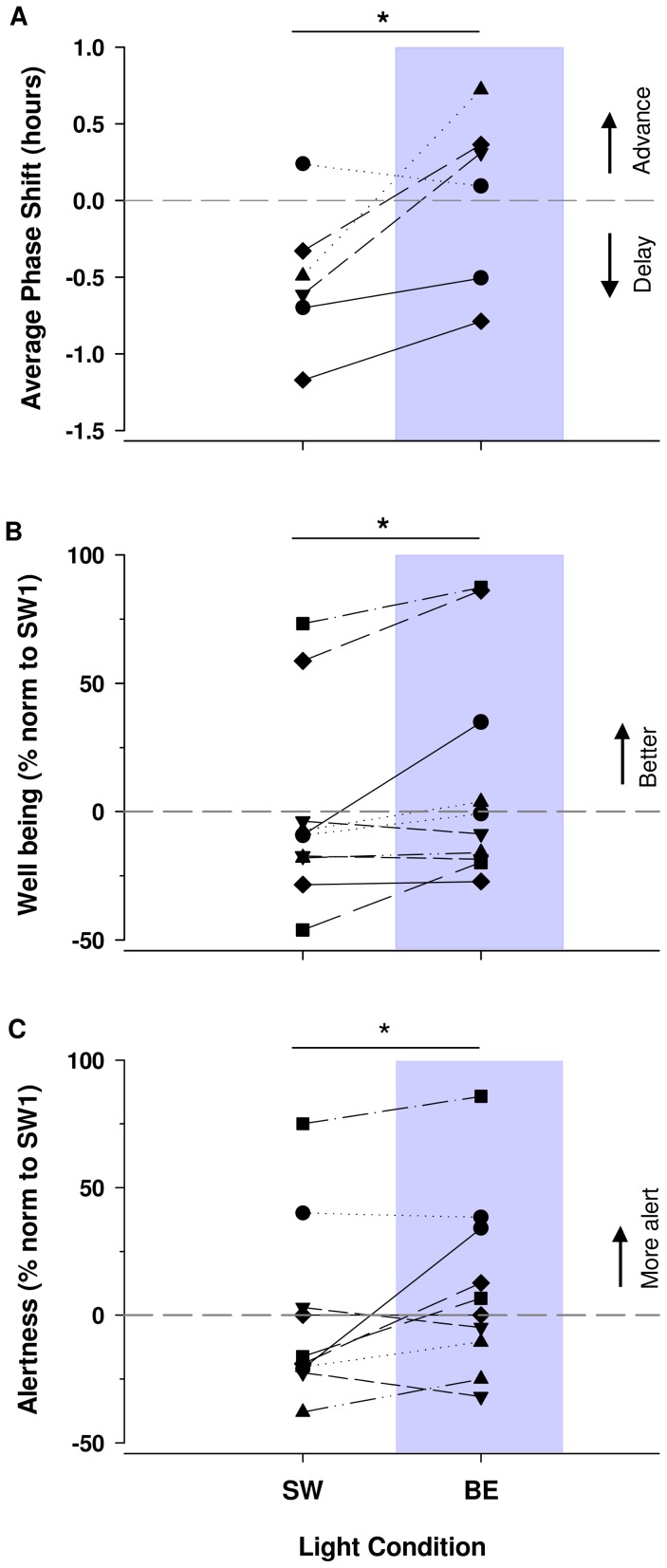
Impact of BE versus SW light on each participant's phase shift, subjective well being and alertness. Results were normalized to week SW1. Circadian phase advances (positive phase shifts) and delays (negative phase shifts) and increases and decreases in well being and alertness were averaged over the weeks of the same light condition (SW versus BE). **A**. On average DLMOs were significantly delayed during SW light weeks compared to BE light weeks (p<0.05). Average subjective well being (**B**) and alertness (**C**) were significantly increased under BE light weeks compared to SW light weeks (p<0.05).

Due to a power outage in our freezer room, salivary melatonin was successfully preserved for only 5 of the 9 weeks of the study in 6 out of 10 subjects (n = 6, 54 DLMOs). DLMO of light exposure weeks (BE3, SW5, BE7, SW9) were compared to baseline week W1. Analyses found no free-running of DLMO under both lighting conditions. No circadian phase delay of melatonin during BE light weeks (−0.005±0.3 h, and −0.008±0.26 h, for weeks BE3 and BE7 respectively). Conversely, a circadian phase delay of −0.64±0.21 h and −0.45±0.34 h was observed during weeks SW5 and SW9 respectively, under SW light conditions (Figure 4. A; Figure S5. A). DLMO were significantly delayed under SW light compared to BE light (p<0.05) (Figure 5. A, Figure S5. B, Table 2). Overall, compared to the initial circadian phase measured during the baseline SW1, SW light elicited 9 phase delays out of 10 phase shifts (Figure S6). Such a results yields that phase delays occurring under SW light weeks were not due to chance (p<0.01), whereas phase delays occurring under BE were occurring in a random fashion (p = ns). Phase angles between DLMO and lights off time were not significantly different under SW and BE light weeks (Table 2).

### Neurobehavioral results

Neurobehavioral data was successfully collected and analyzed in all of the ten participants (n = 10). Our results show a significant effect of “time into the study” on reaction time and motivation. Reaction time as assessed by PVT decreased with the time course of the study (F (1, 9) = 6.38, p<0.05). Subjectively assessed motivation increased throughout the study (F (1, 9) = 8.17, p<0.05). Subjective well being, on the other hand, was significantly increased under BE light compared to SW light (F (1, 9) = 5.31, p<0.05) (Figure 4. B, Figure 5. B). Subjective alertness was also marginally increased across weeks under BE light (p = 0.08) (Figure 4. C). Moreover, there was a significant increase in subjective alertness under grouped BE light weeks compared to grouped SW weeks (p = 0.05) (Figure 5. C). Non-significant fluctuations in subjective mood (p<0.1), time estimation (p = 0.6), motivation (p = 0.1), and KSS scores (p = 0.2), were also observed across the 9 weeks of the study (Figure S7, Figure S8). There was no significant effect of BE light on PVT and stroop tests.

## Discussion

The present study shows that the circadian timing system phase delays in subjects that are chronically (i.e., 2 weeks or more) exposed to modest daytime illuminances (of about 175 lux in the average horizontal angle of gaze) with a correlated color temperature of 4,100K. An exposure to blue-enriched white light (17,000K) of similar duration and illuminance, however, is effective to maintain a stable circadian phase angle of entrainment.

Environmental sunlight varies between 1,000 lux on a heavily overcast day and 100,000 lux with the sun directly overhead. Such light intensities are largely sufficient to successfully entrain the circadian timing system. Most indoor workspaces, however, do not offer such intensities but rather have an illumination that rarely exceeds 300 to 500 lux. In polar regions, entrainment of the circadian system during the winter is almost exclusively dependent on indoor lighting, and is not always achieved due to insufficient light levels or inadequate light spectrum. Therefore, in such extreme environments, optimizing the intensity and spectral composition of ambient light is crucial for optimal physiological and behavioral functioning.

The present study shows a relatively stable rest-activity cycle under SW and BE light, with no effect of BE light on NPCA parameters (amplitude, IS, IV). It is of interest to mention that two out of ten subjects displayed short episodes of free-running of their motor activity under SW but not under BE light. Nevertheless, we insist on the fact that rest activity cycles do not adequately estimate circadian timing and entrainment in humans, since in these two subjects, the free run of actigraphy was not confirmed by a free run of their DLMOs.

Although no effect of BE light was directly observed on the rest-activity pattern, our results show a phase delay of DLMO under SW light, in agreement with previous findings by Kennaway and Van Dorp in 1991 and Mottram and colleagues in 2011 [32], [47]. This circadian phase shift is not observed under the BE light condition. Moreover, across the alternating lighting conditions of the study, the observed circadian phase delay under SW light (SW5, SW9) was counteracted during exposure to blue-enriched white light (BE7). These results are in agreement with data from a recent study by Mottram and colleagues [47] showing that timed blue-enriched light exposure using light boxes can significantly phase advance circadian phase. We estimate that if recurrent 2-week segments of BE light exposure had not be included in our protocol, a free running circadian rhythm would have been observed in some of our participants, as reported in previous studies [32] (for review see [44]). In their study, Kenneway and Van Drop reported that when the sun reappeared during polar spring, circadian entrainment was reestablished for 6-sulfatoxymelatonin, cortisol, sodium, and potassium. In our study, we found that chronic BE light exposure during the daytime was as successful as the spring sun in correcting the circadian drift occurring during polar winter.

Previous studies have failed to report any extra effects of blue-enriched white light on phase shift [48], [49] or treatment of winter depression compared to SW light [50]. In those studies, very high levels of white light (5000 lux–9000 lux) were used as control light, which was likely saturating the response under control condition, and rendering a superior effect of BE almost impossible. In our study, our lighting conditions during daytime were moderate and similar between ambient lighting conditions (SW, average: 160 lux; BE, average: 171 lux). Therefore, aside from protocol differences, the light levels used in different protocols could explain the discrepancies between previous studies and ours.

A delay in sleep and other circadian outputs (melatonin and core body temperature) have been previously reported between polar winter and summer [34], [51]. Previous interventions have been designed to advance circadian phase, such as bright light exposure in the early morning. Trials using 30 minutes or 60 minutes of light exposure in the morning have been successful in advancing the circadian phase and sleep onset, and reducing morning drowsiness [30], [31], [52]. However, with the busy schedule of the personnel of polar stations, 30–60 minutes of light treatment every day in the early morning is not always practical. Our findings show that a paradigm involving a non-timed chronic exposure to an ambient BE light during the daytime offers a practical alternative to a timed exposure to bright light (as in phototherapy approaches) to avoid the delayed circadian phase and sleep loss otherwise observed under normal lighting conditions. It is of importance to indicate that increasing the intensity of SW light might have a similar efficacy as BE light of lower intensity. In fact, this is what we would predict based on the sensitivity of visual and non-visual photoreceptors (Najjar et al. 2014). In this study, we show that at a same illuminance (lux level), or even irradiance (w/m2), a blue enriched fluorescent white light (BE) is more efficacious than a standard fluorescent white light in maintaining circadian entrainment (phase) and alertness and well being.

Our results show a decrease in sleep duration under SW light compared BE light. This sleep loss is the result of a delayed sleep onset, which is not compensated by a delayed wake time. Based on the literature, we expected to find a better sleep efficiency under BE light [36], [40], [47] due to adequate circadian entrainment. Sleep efficiency derived from actigraphy, however, did not reveal any difference between light conditions. It could be that actigraphy is not sufficiently precise detect subtle changes of sleep structure efficiency, or that there is no effect of BE light on this parameter. Nevertheless, we cannot conclude on this point from the current data.

Sub-syndromal seasonal affective disorders (SAD) and depressive symptoms have been reported in Polar Regions [53]–[55]. The present study is the first to assess a relatively large panel of estimates of mood and well being, and neurobehavioral performances in a polar station. Our results show a significant increase in well being and alertness under BE light compared to SW light weeks. These results are in agreement with, and extend, previous findings, showing a positive effect of BE light on subjective measures of sleep quality and cognitive performance in office workers and polar workers [40], [52]. Our results also show that the increase in self-reported well being and alertness under BE light does not dissipate over long periods (9 weeks).

The study however, has a few limitations that should be reported. First, the small number of subjects (n = 6) available for melatonin analysis may have disfavored some statistical significance across different lighting condition weeks. A larger number of subjects is needed to confirm these observations. The use of multiple Antarctic bases or comparable Arctic environments, as suggested by Mottram and colleagues [47], may allow to address these issues. Second, neurobehavioral and DLMO data were very variable. Such variability in response to BE light amongst subjects could be explained by a difference in polymorphism in the clock gene PER3. In fact, light sensitivity in humans may be modulated by a clock gene polymorphism since humans homozygous for the PER3 5/5 allele are particularly more sensitive to blue-enriched light than PER3 4/5 or PER 4/4 individuals [56], [57]. This is unlikely, however, given the low prevalence of PERr3 5/5 in the population (∼10%), but cannot be excluded. The difference in the endogenous period between individuals could also account for such inter-individual variability. In fact, there is a high correlation between circadian phase of melatonin and endogenous circadian period [10]. Therefore, inter-individual differences in periods could account for inter-individual differences in phase angle of entrainment, and consequently melatonin phase. In addition, the closer the endogenous period is to 24 hours, the easier the circadian system can be entrained by light to the light-dark cycle [58]. Therefore, the inter-individual variability observed in the degree of misalignment could also directly be related to individual differences in endogenous periods. There is also evidence for an increase in light sensitivity during the winter in the Antarctic [59]. Unless this increase in sensitivity is wavelength dependent we do not expect it to affect our results. Third, our subjects had knowledge of time, and had scheduled work tasks. This might mask the rest activity rhythm, and explain why actigraphy was not differentially affected by the two light conditions, despite a difference in their effect on circadian synchronization. Knowledge of time, however, is not expected to alter melatonin phase profile, unless combined with different patterns of light exposure. Circadian rhythms have been shown to free run, even when the subjects had knowledge of time, even under relatively high indoor light levels (300 lx to 1000lx) [32]. Overall, despite these limitations, our results are in favor of a beneficial effect of BE light over SW light on the circadian timing system, and neurobehavioral functions.

## Conclusion

We present evidence in this study that, at illuminances of about 175 lux in the average angle of gaze, artificial blue-enriched white light of 17000K during daytime is capable of correcting, or avoiding, circadian phase delay and sleep loss during polar winter that would otherwise occur under regular daytime white light (4100K) of similar illuminance.

We propose that the superior efficacy of blue-enriched white light versus standard white light suggests that melanopsin-based mechanisms are involved in the activation of the non-visual functions studied, and that their responses do not dampen with time (over 9-weeks).

We suggest that blue-enriched white light could be used as a countermeasure to circadian disorders, and well being and alertness decrements in conditions where light levels are chronically low or moderate during the daytime, and solar light not available for extended periods of time, such as in polar base stations, space stations, space class missions, submarines, and workspaces lit solely with artificial light.

## Supporting Information

Figure S1 **Relative spectral sensitivity of the LightWatcher's photodiodes.** The device (Lighwatcher) contains 5 photodiodes with peak sensitivities around: IR (860 nm), red (620 nm), green (540 nm), blue (460 nm) and UV lights (350 nm). This device was used to monitor continuously individual spectral light exposure during the study.
(PDF)

Figure S2 **Raster chart of incident spectral light exposure (in the average angle of gaze) over 8 weeks.** Shown data are a synthesis of measurements collected with LightWatcher personal data loggers from several subjects who were working in comparable light environments. The depicted color values were calculated from the output signals of 3 LightWatcher photodiodes with peak sensitivities around: red (620 nm), green (540 nm), and blue (460 nm), and averaged in 15 minute intervals. Missing data are shown in dark gray color.
(TIF)

Figure S3 **Relative spectral composition of the SW and BE lighting environments.** Whether obtained from manual environmental measurements or from individual proxy of corneal measurements (from data loggers), BE light contained significantly more short wavelength blue light than SW light.
(TIF)

Figure S4 **Average activity under SW light weeks and BE light weeks.** Average daytime and night-time activity was not different between lighting conditions.
(TIF)

Figure S5 **Melatonin phase change in local time.** A. Average DLMO over time, in local time. B. Average DLMO in local time, grouped by light condition. On average DLMOs were significantly delayed during SW light weeks compared to BE light weeks (p<0.05).
(TIF)

Figure S6 **Individual change in circadian phase across the 9 week study.** Notice that SW light elicited 9 phase delays out of 10 phase changes. This was not the case under BE light.
(TIF)

Figure S7 **Impact of light condition on neurobehavioral responses over 9 weeks.** Results are normalized to week SW1, and are expressed as means ± SE. Non-significant fluctuations in average sleepiness (A), time estimation (B), motivation (C), and subjective mood (D), are observed across the nine weeks.
(TIF)

Figure S8 **Impact of BE versus SW light on each participant's motivation, mood and sleepiness.** Results are normalized to week SW1, and are expressed as mean ± SE. Increases and decreases in motivation, mood and sleepiness compared to baseline SW1 are plotted against the combined weeks of same light condition. Although a trend was apparent, on average, there was no significant effect of light on motivation (**A**) mood (**B**) and sleepiness (**C**). Average mood was marginally increased under BE light compared to SW light (**B**) (p<0.1, §).
(TIF)

Figure S9 **Raster plot of actigraphy of the 4 participants, S7 (A), S8 (B), S6 (C) S1 (D).** Plots show a relatively stable rest-activity pattern under both lighting conditions. Participant (code S8, **B**) displayed a large delay of activity on SW9. Melatonin secretion of S8, on the other hand, was not free-running (*).
(TIF)

Figure S10 **Raster plot of activity of the 4 participants, S3 (A), S4 (B), S5 (C), S8 (D).** Plots showing a variation in the phase of the rest activity pattern. Subject (code S5) (**C**) had a saturation of activity due to a lower sensitivity threshold of the actiwatch.
(TIF)

Table S1 **Lighting characteristics of the Concordia polar station (Dome C), as determined via irradiance measurements at multiple locations.**
(PDF)
