# Supplementary material for: Chronic Artificial Blue-Enriched White Light Is an Effective Countermeasure to Delayed Circadian Phase and Neurobehavioral Decrements
Source: PLoS One. 2014 Jul 29;9(7):e102827. doi: 10.1371/journal.pone.0102827 (PMC4114570; doi:10.1371/journal.pone.0102827)
Supplement: Figure S1 — Relative spectral sensitivity of the LightWatcher's photodiodes. The device (Lighwatcher) contains 5 photodiodes with peak sensitivities around: IR (860 nm), red (620 nm), green (540 nm), blue (460 nm) and UV lights (350 nm). This device was used to monitor continuously individual spectral light exposure during the study. (PDF) [file pone.0102827.s001.pdf]

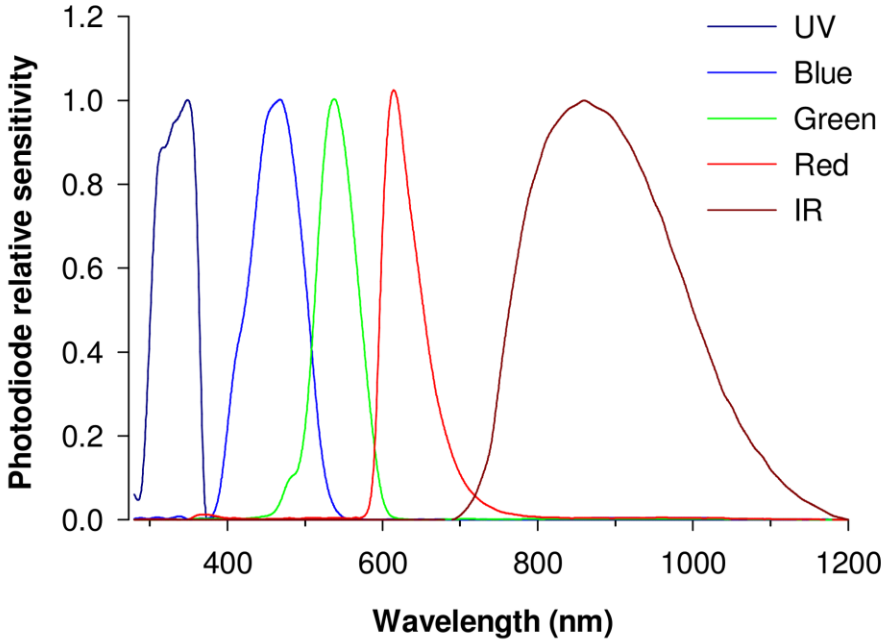

Figure S1. Relative spectral sensitivity of the LightWatcher's photodiodes. The device (Lighwatcher) contains 5 photodiodes with peak sensitivities around: IR (860 nm), red (620 nm), green (540 nm), blue (460 nm) and UV lights (350 nm). This device was used to monitor continuously individual spectral light exposure during the study.
