# Supplementary figures and images for: Chronic Artificial Blue-Enriched White Light Is an Effective Countermeasure to Delayed Circadian Phase and Neurobehavioral Decrements
Source: PLoS One. 2014 Jul 29;9(7):e102827. doi: 10.1371/journal.pone.0102827 (PMC4114570; doi:10.1371/journal.pone.0102827)

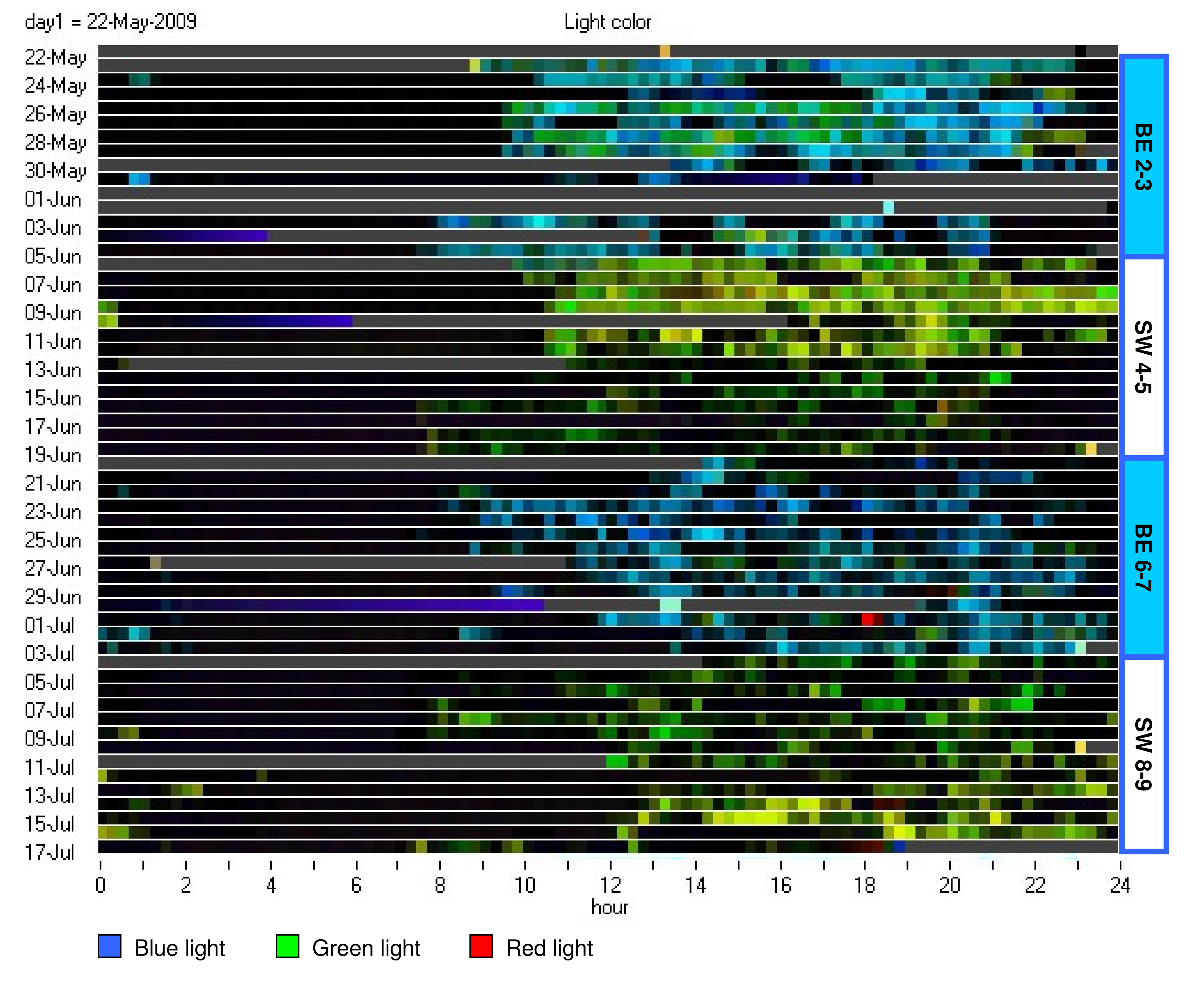

Supplement: Figure S2 — Raster chart of incident spectral light exposure (in the average angle of gaze) over 8 weeks. Shown data are a synthesis of measurements collected with LightWatcher personal data loggers from several subjects who were working in comparable light environments. The depicted color values were calculated from the output signals of 3 LightWatcher photodiodes with peak sensitivities around: red (620 nm), green (540 nm), and blue (460 nm), and averaged in 15 minute intervals. Missing data are shown in dark gray color. (TIF) [file pone.0102827.s002.tif]

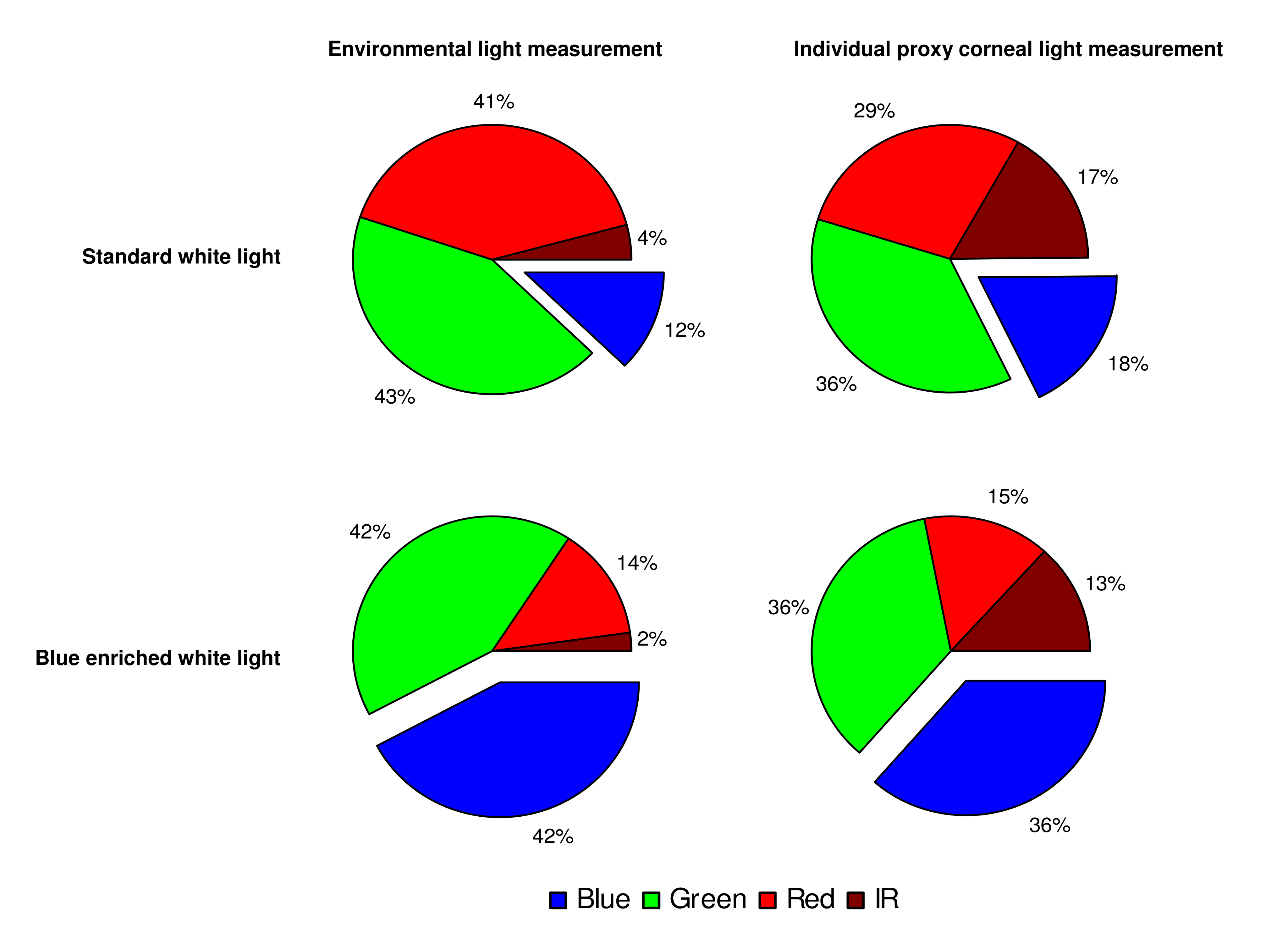

Supplement: Figure S3 — Relative spectral composition of the SW and BE lighting environments. Whether obtained from manual environmental measurements or from individual proxy of corneal measurements (from data loggers), BE light contained significantly more short wavelength blue light than SW light. (TIF) [file pone.0102827.s003.tif]

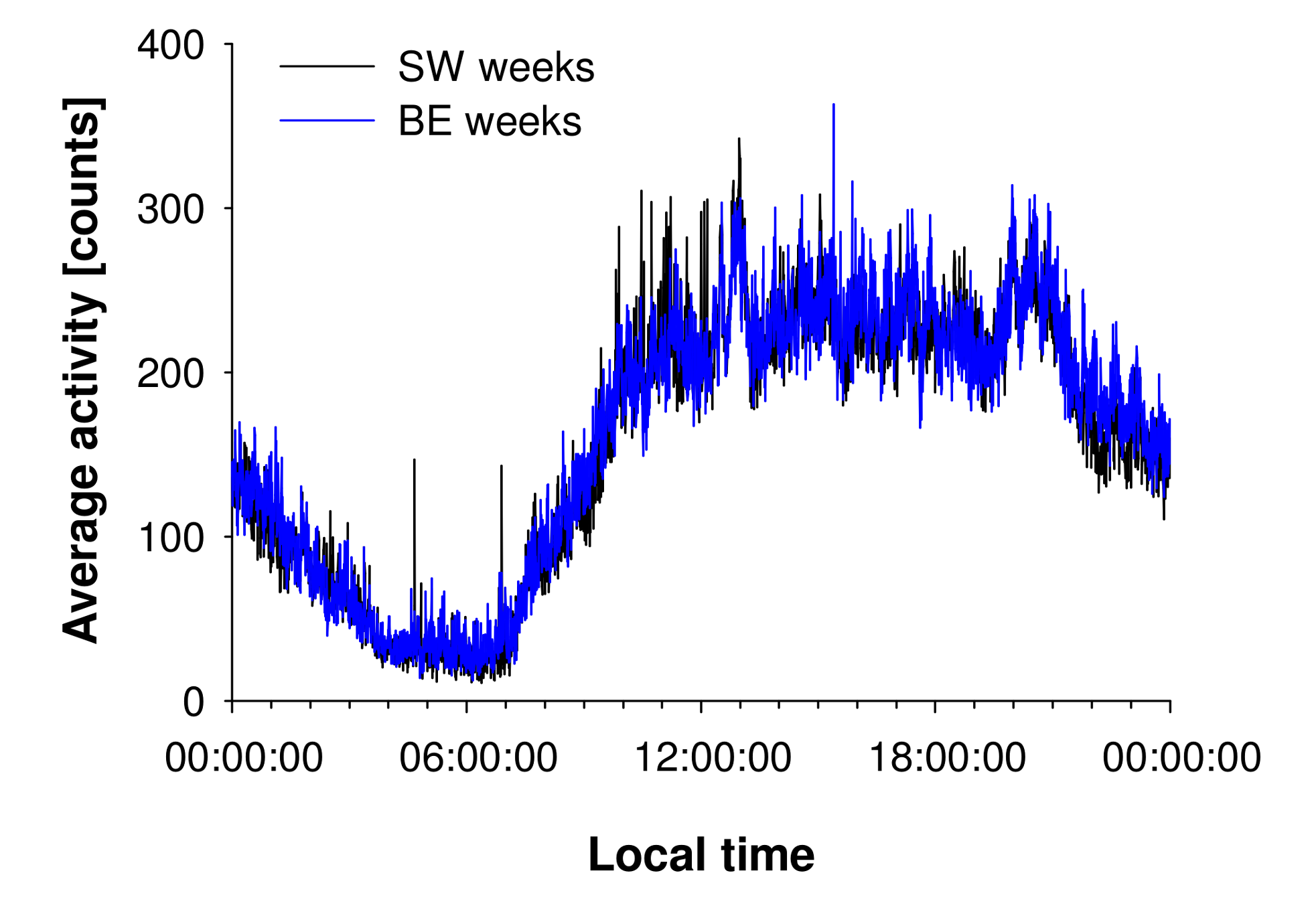

Supplement: Figure S4 — Average activity under SW light weeks and BE light weeks. Average daytime and night-time activity was not different between lighting conditions. (TIF) [file pone.0102827.s004.tif]

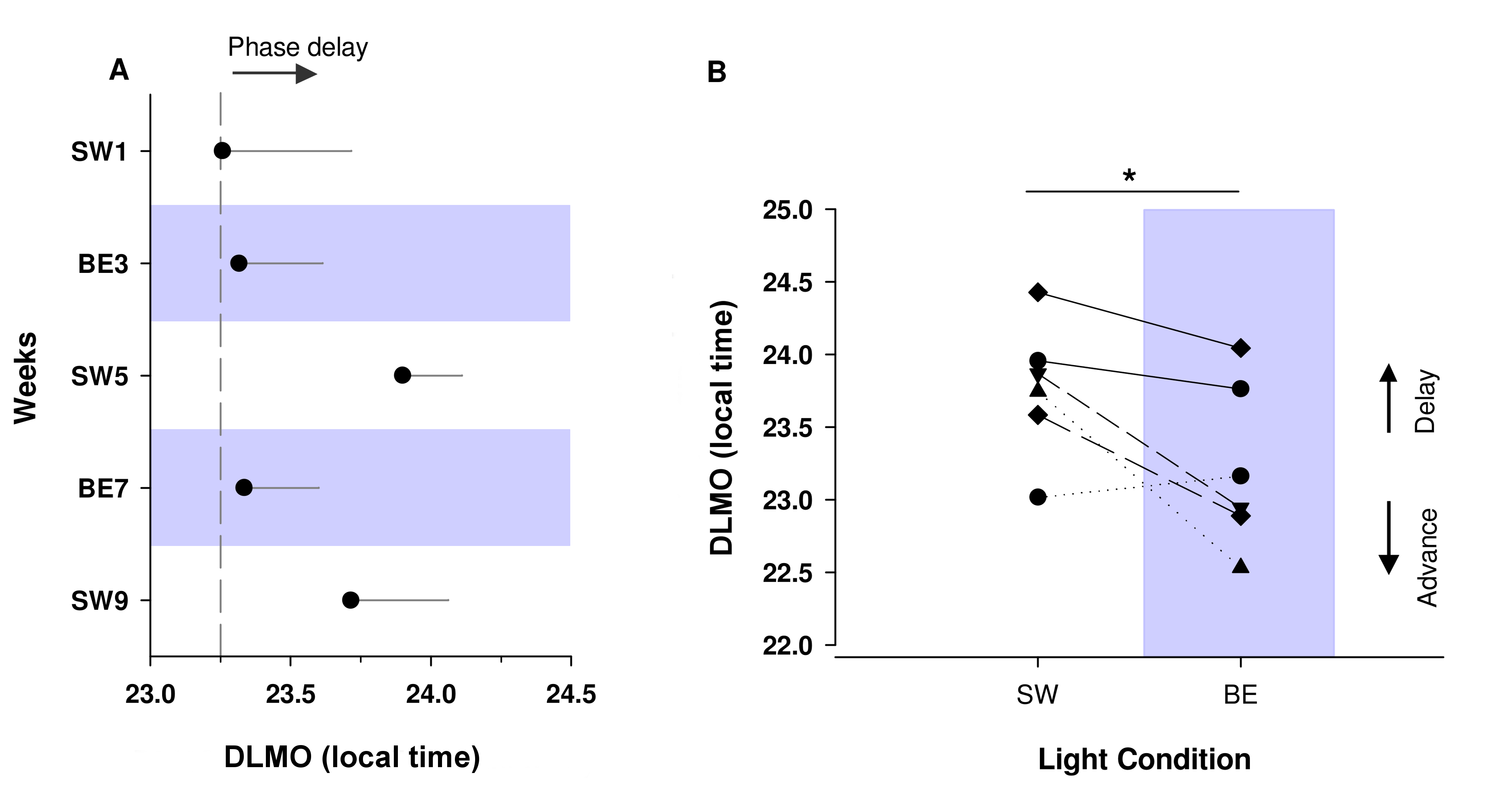

Supplement: Figure S5 — Melatonin phase change in local time. A. Average DLMO over time, in local time. B. Average DLMO in local time, grouped by light condition. On average DLMOs were significantly delayed during SW light weeks compared to BE light weeks (p<0.05). (TIF) [file pone.0102827.s005.tif]

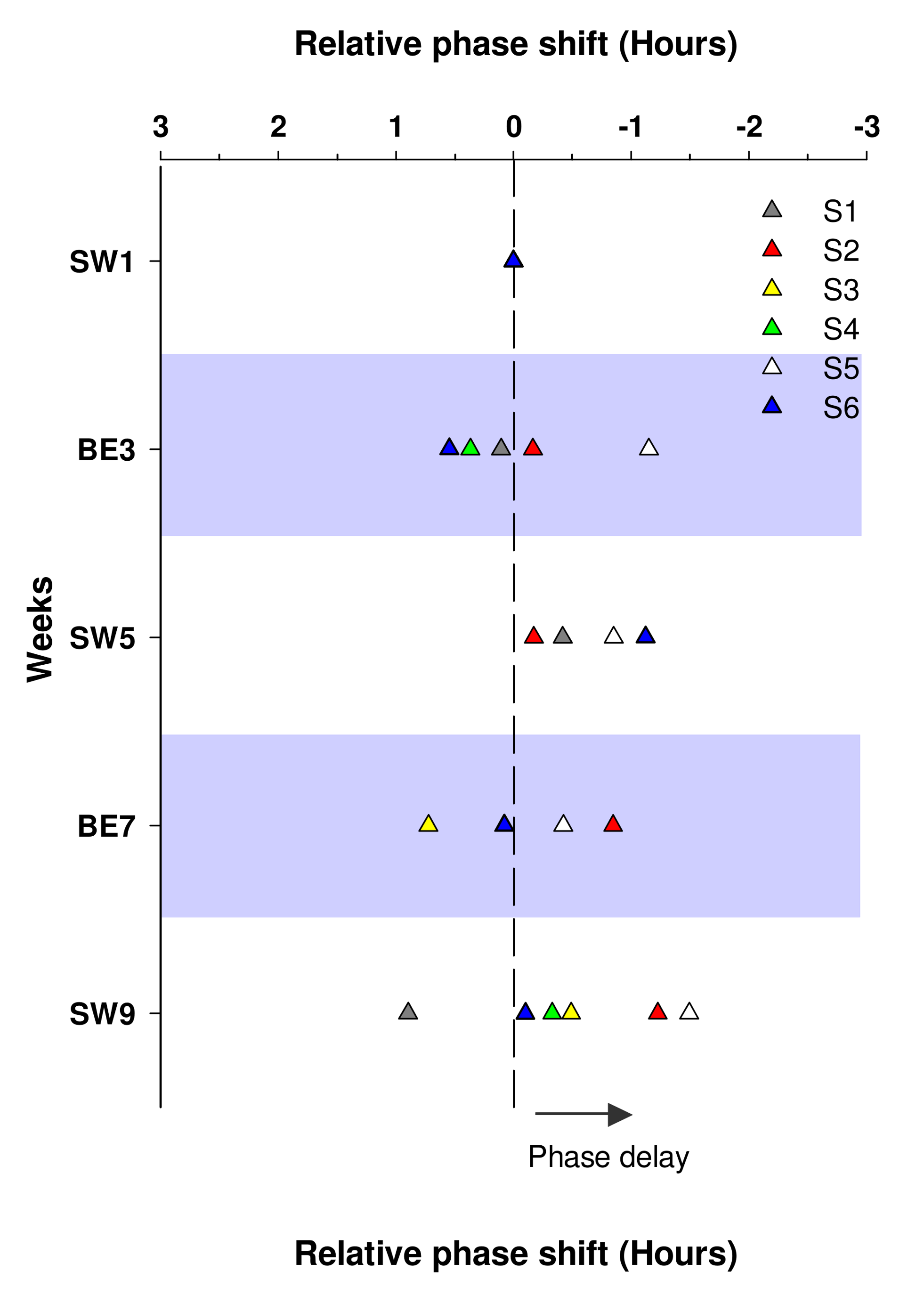

Supplement: Figure S6 — Individual change in circadian phase across the 9 week study. Notice that SW light elicited 9 phase delays out of 10 phase changes. This was not the case under BE light. (TIF) [file pone.0102827.s006.tif]

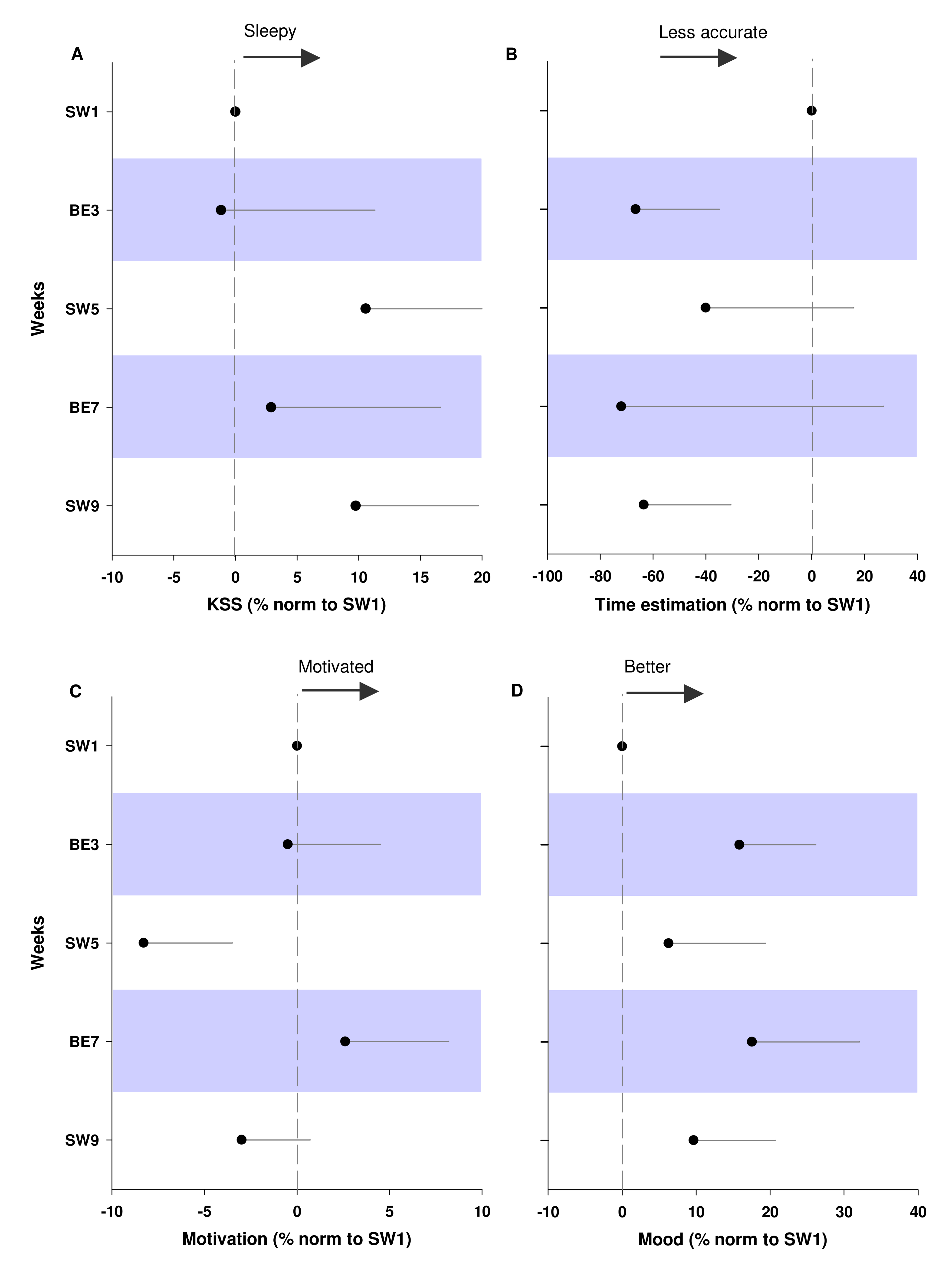

Supplement: Figure S7 — Impact of light condition on neurobehavioral responses over 9 weeks. Results are normalized to week SW1, and are expressed as means ± SE. Non-significant fluctuations in average sleepiness (A), time estimation (B), motivation (C), and subjective mood (D), are observed across the nine weeks. (TIF) [file pone.0102827.s007.tif]

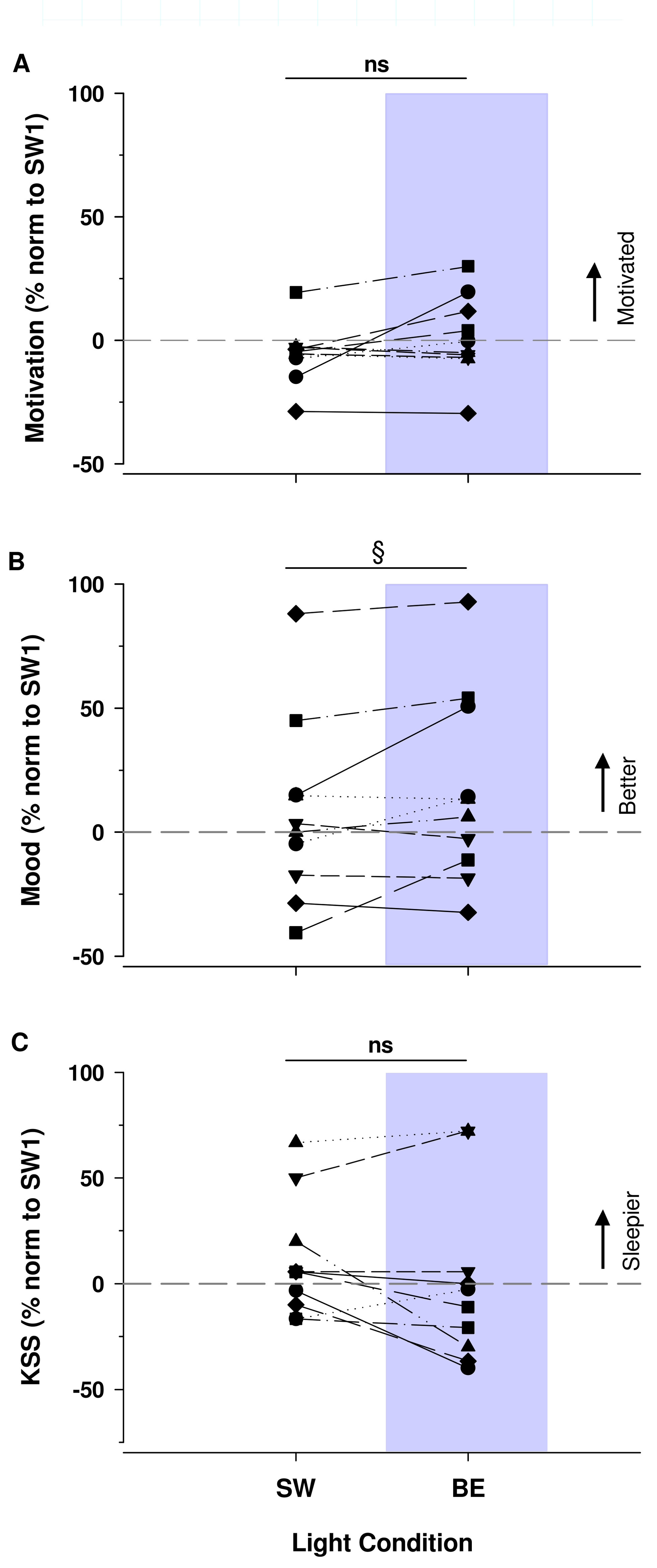

Supplement: Figure S8 — Impact of BE versus SW light on each participant's motivation, mood and sleepiness. Results are normalized to week SW1, and are expressed as mean ± SE. Increases and decreases in motivation, mood and sleepiness compared to baseline SW1 are plotted against the combined weeks of same light condition. Although a trend was apparent, on average, there was no significant effect of light on motivation (A) mood (B) and sleepiness (C). Average mood was marginally increased under BE light compared to SW light (B) (p<0.1, §). (TIF) [file pone.0102827.s008.tif]

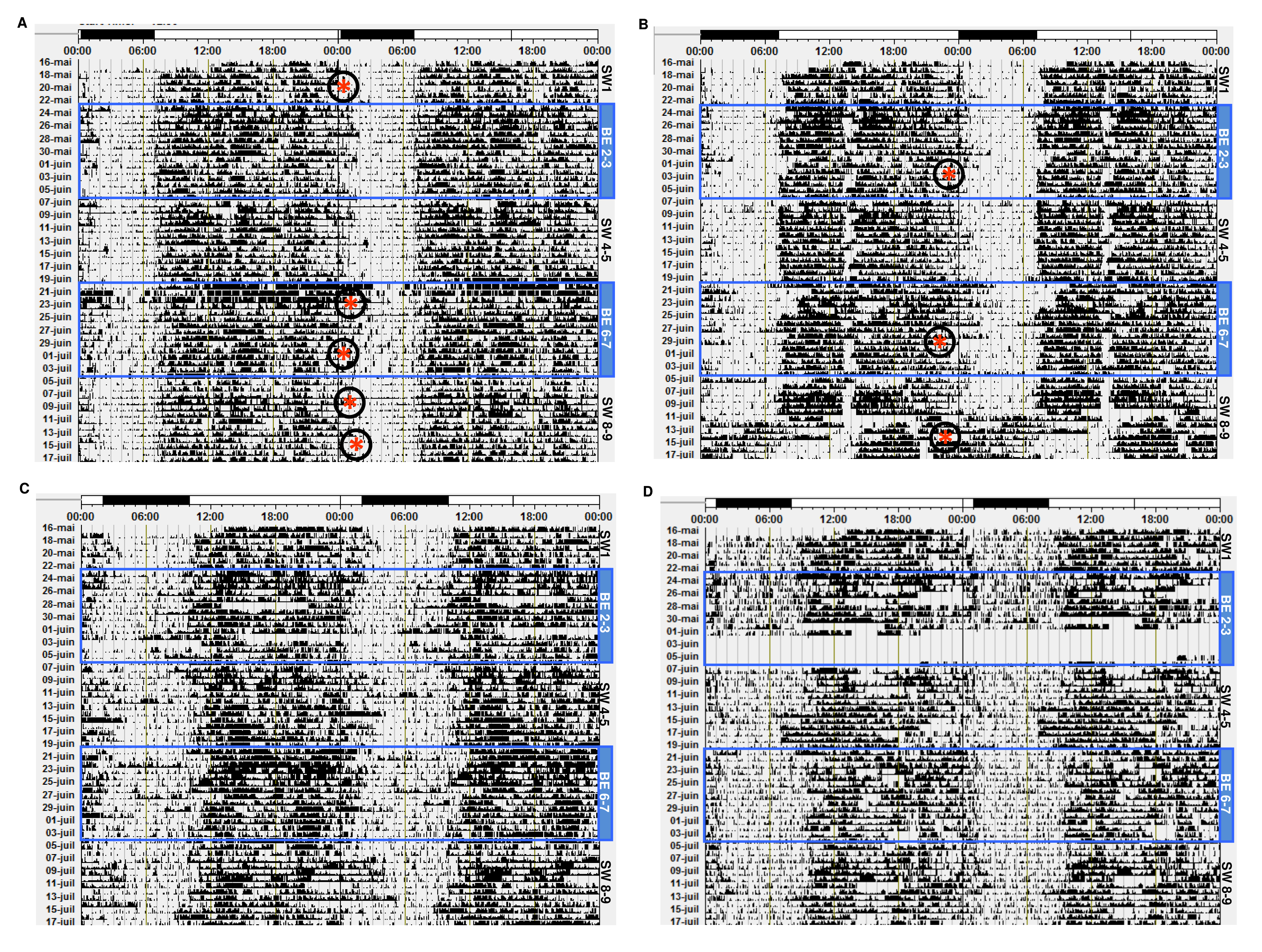

Supplement: Figure S9 — Raster plot of actigraphy of the 4 participants, S7 (A), S8 (B), S6 (C) S1 (D). Plots show a relatively stable rest-activity pattern under both lighting conditions. Participant (code S8, B) displayed a large delay of activity on SW9. Melatonin secretion of S8, on the other hand, was not free-running (*). (TIF) [file pone.0102827.s009.tif]

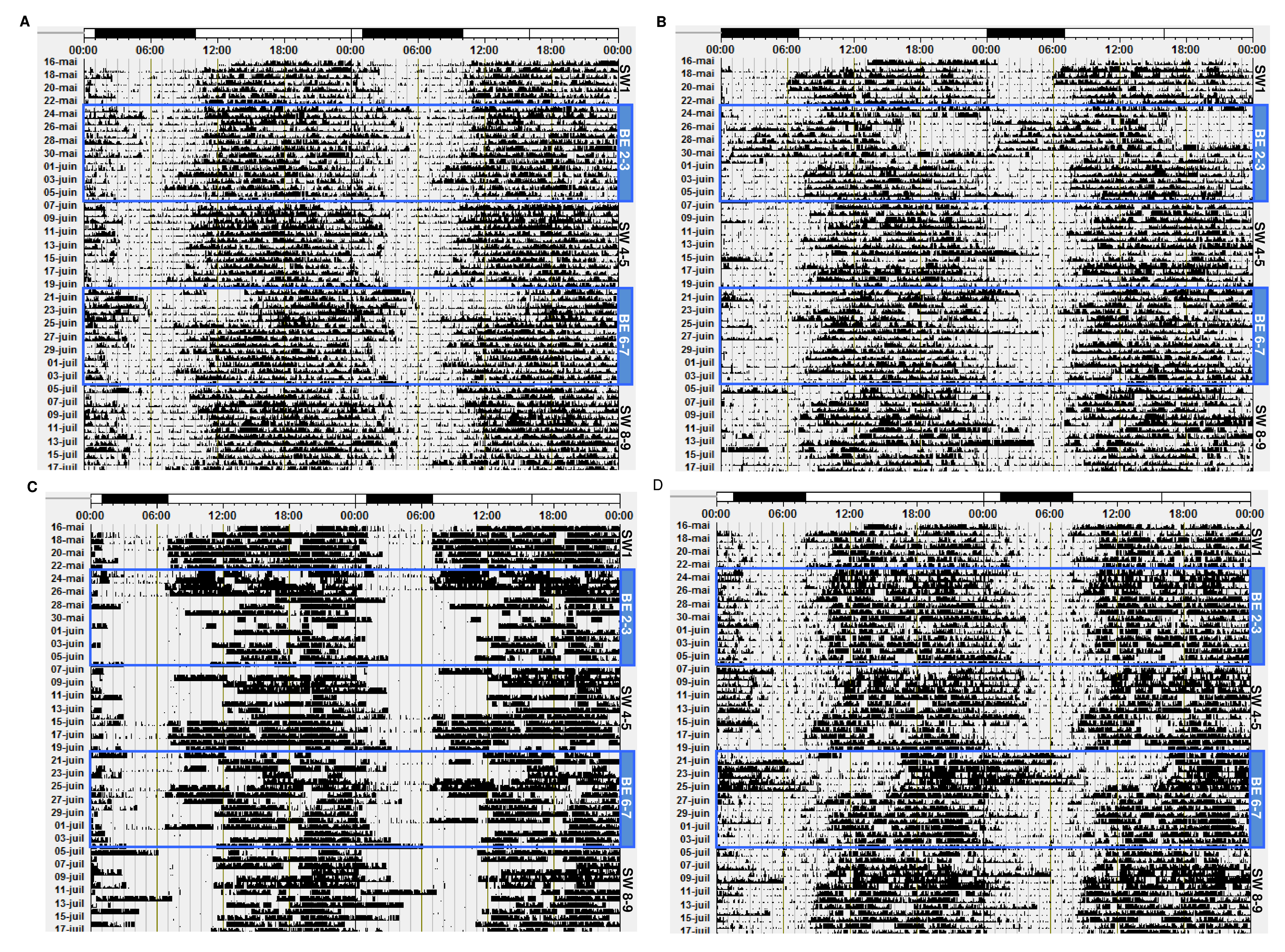

Supplement: Figure S10 — Raster plot of activity of the 4 participants, S3 (A), S4 (B), S5 (C), S8 (D). Plots showing a variation in the phase of the rest activity pattern. Subject (code S5) (C) had a saturation of activity due to a lower sensitivity threshold of the actiwatch. (TIF) [file pone.0102827.s010.tif]
