# Supplementary material for: Chronic Artificial Blue-Enriched White Light Is an Effective Countermeasure to Delayed Circadian Phase and Neurobehavioral Decrements
Source: PLoS One. 2014 Jul 29;9(7):e102827. doi: 10.1371/journal.pone.0102827 (PMC4114570; doi:10.1371/journal.pone.0102827)
Supplement: Table S1 — Lighting characteristics of the Concordia polar station (Dome C), as determined via irradiance measurements at multiple locations. (PDF) [file pone.0102827.s011.pdf]

Table S1. Lighting characteristics of the Concordia polar station (Dome C), as determined via irradiance measurements at multiple locations

	Irradiance mean values (mW/m²/s)							
	Blue		Green		Red		IR	
**Concordia locations**	**SW**	**BE**	**SW**	**BE**	**SW**	**BE**	**SW**	**BE**
Astro Lab1	1.6	6.2	5.5	6.0	5.8	2.1	0.3	0.3
Astro Lab2	0.9	2.9	2.7	2.8	2.8	1.0	0.2	0.2
Atmos Lab	1.5	6.1	4.9	5.7	4.4	1.8	0.3	0.2
Female Bathroom	0.9	7.0	3.8	7.0	3.8	1.9	0.4	0.2
Male Bathroom	1.5	7.5	4.0	7.0	2.6	1.9	0.3	0.2
S1 Bedroom	0.6	3.0	1.7	2.8	1.7	0.9	1.6	1.2
Biomedical Lab	1.7	7.6	6.9	7.7	6.4	3.0	0.3	0.3
Bureau Technique Dome C	1.3	7.5	5.4	7.3	5.4	2.2	0.3	0.2
Consultation	1.5	9.3	6.3	8.2	6.5	2.3	0.4	0.2
Corridor 1	1.3	3.7	3.8	5.5	2.9	2.7	0.3	0.2
Corridor 3 calm	1.7	8.5	6.9	9.1	7.1	3.8	0.5	0.4
Corridor 3 noisy	1.0	5.2	3.3	5.3	3.4	2.2	1.1	0.4
Doc Bedroom	1.1	3.6	3.5	3.4	3.6	1.2	0.4	0.5
GlacioLab	1.3	8.1	5.2	7.8	5.5	2.4	0.3	0.3
Grey Water Treatment Unit	1.3	6.8	5.7	6.6	5.5	1.5	0.4	0.2
Kitchen	1.3	6.9	4.9	6.9	4.3	1.9	0.4	0.4
S2 Bedroom	1.1	4.5	3.2	4.3	3.1	1.4	0.3	0.1
Salle Manger	1.0	5.9	3.8	5.8	3.8	1.7	0.3	0.4
Salon	1.0	5.8	3.7	5.7	3.8	1.6	0.3	0.3
Seismo Lab	1.2	5.8	5.1	5.7	5.2	1.8	0.3	0.2
Usine	1.3	4.1	3.3	4.1	2.3	1.2	0.3	0.2
Vaisselle	1.5	4.8	4.2	4.8	2.8	1.3	0.3	0.3
